# Healing spaces: a retrospective cohort study on the effect of outdoor spaces in psychiatric inpatient units on PRN medication use, seclusion/restraints, and constant observation

**DOI:** 10.3389/fpsyt.2025.1731925

**Published:** 2026-01-15

**Authors:** Kevin Liu, Ema Saito, Howard Linder

**Affiliations:** 1 Donald and Barbara Zucker School of Medicine at Hofstra/Northwell, Hempstead, NY, United States; 2 Department of Psychiatry, The Zucker Hillside Hospital, Northwell Health, Glen Oaks, NY, United States

**Keywords:** acute psychiatry, constant observation, outdoor space, PRN medication, restraint, seclusion

## Abstract

**Background:**

This retrospective chart review investigated the differential correlation of outdoor space accessibility with as-needed(PRN) medication use, constant observation(CO), and seclusion/restraint across a geriatric psychiatry unit (Unit A) and a general psychiatry unit (Unit B). While the influence of psychiatric facility design on patient aggression has been explored, empirical investigation specifically into the correlation of outdoor space accessibility with aggression in psychiatric inpatients, remains limited.

**Methods:**

We compared the use of oral and intramuscular PRN medications, seclusion/restraint, and CO during periods of outdoor space accessibility (2022-2023) versus inaccessibility (2024). Outdoor space was inaccessible for Unit A from January 31 to April 18, 2024, and for Unit B from February 12 to May 6, 2024. The analysis was conducted assuming no other significant confounding changes in unit operations or patient populations during the study period. A single factor ANOVA analysis was chosen, but to control the family-wise error rate due multiple comparisons, a Bonferroni correction was applied.

**Results:**

On unit B, outdoor space inaccessibility was significantly associated with increased IM PRN use (mean difference = 1.57 orders/day, 95% CI [0.81, 2.33]), increased seclusion and restraint (mean difference = 0.63 orders/day, 95% CI [0.35, 0.91]), and decreased CO (mean difference = 0.40 orders/day, 95% CI [0.17, 0.63]), with no significant change in PO PRN. On unit A, outdoor space inaccessibility was associated with an increase in PO PRN use (mean difference = 0.64 orders/day, 95% CI [0.26, 1.02]), while IM PRN, seclusion and restraint, and CO did not show any statistically significant increase.

**Conclusion:**

The loss of accessible outdoor space appears to influence general adult psychiatric patients more broadly, contributing to an increased reliance on restrictive interventions. This is hypothesized to be due to reduced opportunities for self-regulation, personal space, and distancing from stressors. Staff perceptions of limited environmental de-escalation options may also play a role in intervention use. Geriatric patients, due to their inherent lower mobility and activity levels, showed an increase in PO PRN use. These findings underscore the potential role of accessible outdoor spaces in promoting patient well-being and potentially minimizing the need for coercive measures in acute psychiatric settings.

## 1 Introduction

Patient aggression in psychiatric hospitals is a problem that poses significant challenges to patient and staff safety. Aggressive behavior is a frequent cause for restraint, seclusion, or constant observation, yet these safety measures do not provide therapeutic value for the patient (1). Restraints and seclusion in particular are subject to controversy due to its potentially traumatizing nature and are only to be used as a last resort when less restrictive measures fail to address a crisis (2). Meanwhile, healthcare workers experience more anger, anxiety, guilt, and burn-out associated with incidents of aggression (1). The development of aggression in psychiatric patient is a complex phenomenon that involves the interplay between patient, clinician, healthcare system, and environmental factors (1). By investigating these factors to reduce aggression, we can improve the well-being of both patients and healthcare workers.

Environmental design of psychiatric facilities has been proven to be valuable for both improving patient recovery and reducing burden on staff (3–5). A 2018 study by Ulrich et al. developed a conceptual model that demonstrates how stress-reducing design features (such as personal space, communal space, noise reduction, windows, art, and gardens) could reduce patient aggression (6). Another study by Jovanović et al. exploring elements of hospital built environments found visiting rooms and mixed-sex wards improved patient satisfaction (7). However, facility design is very multifactorial, and existing literature has found mixed support for the influence of structural design on psychiatric patient aggression (8). Thus, it is important to continue to elucidate how specific design elements relate to aggressive behavior.

Among stress-reducing design elements, outdoor space has been highlighted for positive effects on mental health outcomes. There is a well understood relationship of how features of outdoor spaces such as natural light and connection to nature improve physical and mental well-being as well as reduce stress (3, 9). Outside of healthcare, neighborhood green space has been shown to improve mental health and reduce aggressive behavior in communities (10, 11). Interviews conducted with psychiatric healthcare workers have reported strong support for access to nature (12). While some studies have examined the influence of secure open spaces on staff assaults (e.g (13).), the empirical investigation of how accessible outdoor space specifically correlates with PRN medication use and restrictive interventions in acute psychiatric settings, and its differential influence across patient populations, remains an important research gap. This study aims to address this specific gap. We hypothesized that sustained access to outdoor space would be associated with a decrease in PRN medication use and restrictive interventions.

## 2 Methods

### 2.1 Study design and setting

This study was performed at a free-standing psychiatric hospital in the northeast of the United States. The patient population consists of those who require acute care psychiatric treatment for safety reasons. This includes patients who pose a danger to themselves or others or are unable to care for themselves due to the severity of their psychiatric condition. Unlike in other geographic locations, access to outdoor space during an acute inpatient psychiatric hospitalization is not a requirement in our area with many facilities not having an outdoor area for admitted to acute inpatient psychiatric units. We performed a retrospective cohort study using anonymous information obtained from electronic medical records of patients. This study utilized a quasi-experimental design, comparing outcomes during periods of outdoor space accessibility (control periods) versus inaccessibility (experimental periods) on the same units, effectively acting as a natural experiment due to facility renovations. This study was approved by the institutional research board. All patient data collected was anonymized without any patient identifying information, therefore waiving need for patient consent.

We retrieved data from two inpatient psychiatric units, Unit A and Unit B, in the years 2022, 2023, and 2024. Unit A is a geriatric unit and Unit B is a general adult unit. These units typically have access to outdoor space by means of a spacious open-air patio. This open space is 1330 sq ft on unit A and 1900 sq ft on unit B, featuring a basketball hoop (on the adult unit), ping pong table (on the adult unit), planters with native shrubs and flowers, and shaded seating areas with picnic-like tables and benches. It is enclosed by high fences, providing a secure environment, and staff are present during patient access. Access was generally available during daylight hours when weather permitted, and staffing levels allowed. However, these two units had outdoor space temporarily closed due to renovations in 2024, from Jan 31 to Apr 18 for Unit A, and from Feb 12 to May 6 for Unit B. We extracted variables such as orders for oral (PO) as needed (PRN) sedative or antipsychotic medication, orders for intramuscular (IM) as needed sedative or antipsychotic medication, orders for seclusions and restraints, and orders for constant observation. These variables were chosen as indicators of patient agitation and the need for intervention, serving as proxy measures given the absence of routinely collected direct measurements of aggressive behavior in the EMR. PRN medication, seclusion and restraints, and constant observation are all techniques that are frequently used for immediate management of inpatient aggression (14). While direct measures of aggressive incidents (e.g., frequency and severity scores) would offer greater specificity, these clinical practice proxy measures could be used as indicators of acute behavioral disturbance requiring intervention and were the only consistently available data in the EMR for this retrospective study. We compared these proxy measures in cohorts during the time periods in 2024, which had no outdoor space access, to that in cohorts in the same units during the control periods in 2022 and 2023, which retained outdoor space access.

### 2.2 Variables and data sources

The primary exposure variable was outdoor space accessibility (accessible in 2022 and 2023; inaccessible in 2024). Outcome variables, serving as proxy measures for patient agitation and the need for intervention, included the daily number of orders for PO PRN sedative or antipsychotic medication, IM PRN sedative or antipsychotic medication, seclusion and restraint (combined), and constant observation (CO). Data for these variables were extracted from electronic medical records. Patient demographic data (age, gender, race/ethnicity), legal status on admission, and primary psychiatric diagnoses were also extracted from the EMR for each participant cohort. Primary diagnoses were based on the treating clinicians’ assessments and documentation, consistent with DSM criteria. However, for analysis purposes the diagnoses were group into broader DSM chapter categories.

### 2.3 Population and sample size

The study included all patients admitted to Unit A and Unit B during the specified experimental and control periods (2022, 2023, 2024), resulting in a total of 430 patient admissions across the cohorts. The sample size was therefore determined by the complete census of patient records available during these defined periods, rather than an *a priori* power calculation. No patients admitted during these periods were excluded from this retrospective study.

### 2.4 Analysis

For each outcome variable (PO PRN, IM PRN, seclusions and restraints, CO), we identified the number of orders per day per unit. Them we compared the average number of orders per day during the experimental time period versus that of the control. We compared the experimental time periods, Jan 31-Apr 18, 2024 for Unit A and Feb 12-May 6, 2024 for Unit B, to two different control time periods of the same date range in 2023 or 2022.

A single factor ANOVA analysis was chosen to compare means across three independent groups (the three calendar years) with an overall significance level of *a* = 0.05. To control the family-wise error rate due multiple comparisons, a Bonferroni correction was applied, where adjusted significance threshold was calculated by dividing *a* by 4 for the number of comparisons per unit. Therefore, the adjusted significance threshold was *a_bonferroni_* = 0.0125, where results were considered statistically significant if *p* was below *a_bonferroni_*. For *post-hoc* analysis of significant ANOVA results, a Tukey-Kramer honest significant difference (HSD) test was performed, with an α = 0.05 for critical q values, to identify the statistical significance in pairwise comparisons of groups while controlling for the family-wise error rate within each ANOVA.

Assessment of Group Comparability and Data Distribution: Prior to performing ANOVA, the comparability of demographic and diagnostic characteristics between the yearly cohorts within each unit was assessed descriptively. Homogeneity of variances was checked through inspection of variance values and not found to be a violation that significantly impacted the ANOVA results, though some differences were noted and reported in the tables. Visual inspection of histograms and Q-Q plots of the residuals for each outcome variable suggested reasonable approximation of normality, or that the sample sizes were sufficiently large for the central limit theorem to apply, minimizing concerns about departures from normality for ANOVA.

## 3 Results

### 3.1 Participant demographics and group comparability

A total of 430 patients were collectively identified across Unit A and Unit B during the experimental and control time periods (2022, 2023, and 2024). All patients were adults, above the age of 18. Both units have a maximum daily census of 20 patients. This study included 223 men (51.9%) and 207 women (48.1%). **Table 1** provides detailed demographic data (age, gender, race/ethnicity) and primary psychiatric diagnoses for each unit and year, as well as combined totals. Given that Unit A was a geriatric psychiatry unit while Unit B was a general psychiatry unit, significant demographic differences were observed between units in age, primary diagnosis, and race/ethnicity, as expected. Within each unit, however, the demographic and diagnostic distributions between the 2022, 2023, and 2024 cohorts were generally stable, supporting the assumption of comparable patient populations across the study periods for each unit, with some minor shifts noted (e.g., in depression diagnoses in Unit A). Some subgroups, particularly within Unit A, had relatively small numbers of patients for specific diagnoses, which should be considered when interpreting those specific results.

**Table 1:** Prevalence of demographic variables and primary psychiatric diagnosis.

Demographic characteristics	Unit A (geriatric psychiatry)			Unit B (general psychiatry)			Combined
Year	2022	2023	2024	2022	2023	2024	
Number	66	52	53	85	86	88	430
Age (average)	68.9	69.6	70.7	36.8	36.4	38.9	53.6
Gender							
Male	28	23	24	49	48	51	223 (51.9%)
Female	38	29	29	37	38	36	207 (48.1%)
Legal Status							
Voluntary	21	15	16	26	26	26	130 (30.2%)
Involuntary	45	37	37	59	60	62	300 (69.8%)
Race and ethnicity							
White	42	34	37	31	30	33	207 (48.1%)
Black	6	8	5	28	32	25	104 (24.2%)
Asian	3	4	3	6	9	9	34 (7.9%)
Multiracial or other	12	6	6	17	13	16	70 (16.3%)
Unknown	2	0	3	3	2	5	15 (3.5%)
Hispanic ethnicity	11	6	4	10	7	9	47 (10.9%)
Primary diagnosis							
Psychosis	11	13	10	44	46	49	173 (40.2%)
Bipolar	3	6	7	8	9	11	44 (10.2%)
Depression	42	25	25	11	13	10	176 (40.9%)
Anxiety	1	2	3	2	3	3	14 (3.3%)
Dementia	9	6	8				23 (5.3%)

### 3.2 PO PRN orders

**Figure 1:**
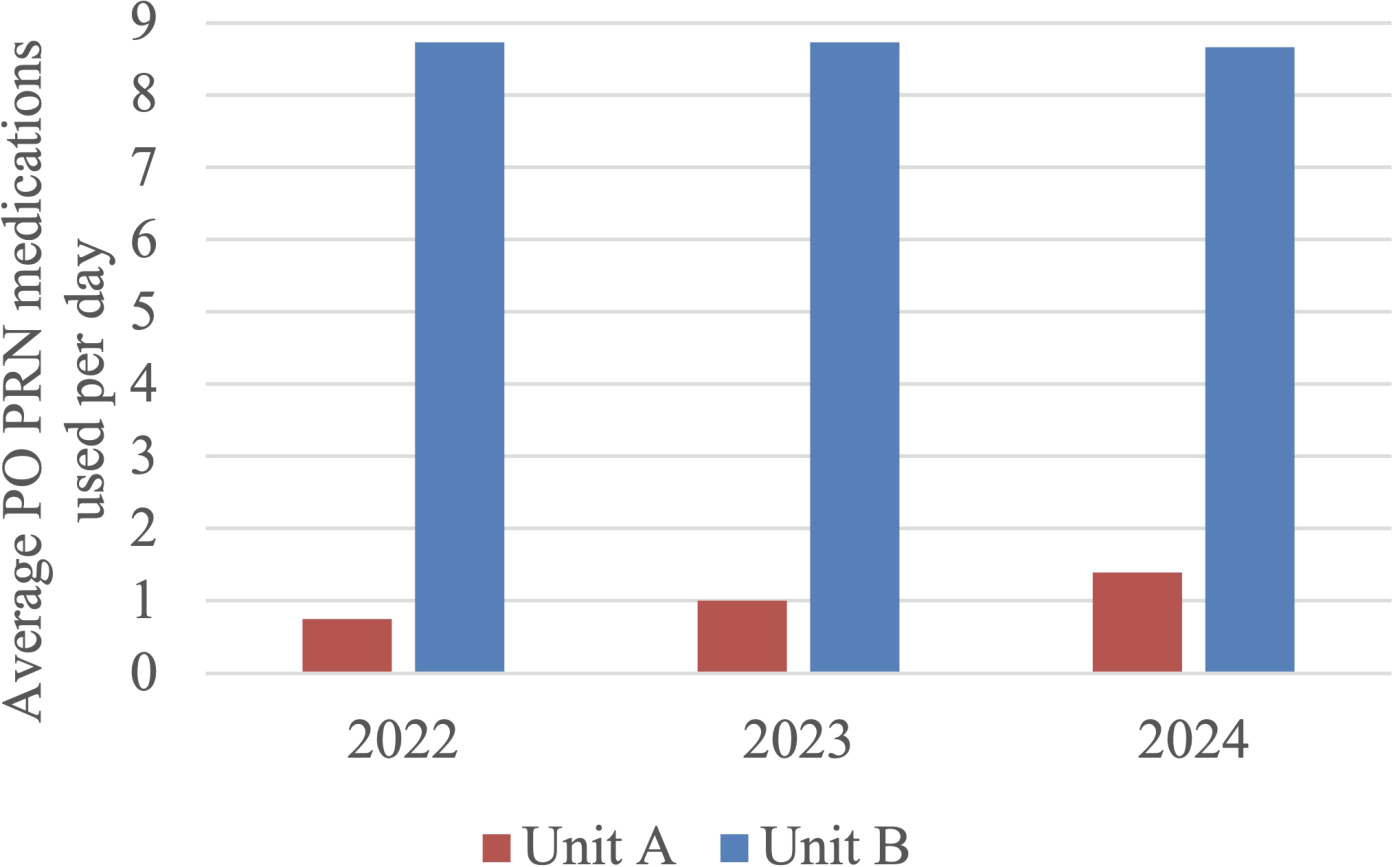
Average PO PRN medications ordered per day in Unit A and Unit B. In Unit A, the 2024 group saw increased PO PRN orders compared to previous years. In Unit B, no trend was observed.

Regarding PO PRN medication orders, ANOVA analysis found a statistically significant difference between groups in Unit A (F = 7.185, p < 0.001), but no significant difference in Unit B (F = 0.008, p = 0.992). Within Unit A, the Tukey-Kramer HSD test (critical q value = 3.337) found a statistically significant increase in PO PRN medication orders when comparing the 2022 vs 2024 (mean difference = 0.636 orders/day, 95% CI [0.255, 1.017], q = 5.310), but not when comparing 2022 vs 2023 (mean difference = 0.244 orders/day, 95% CI [-0.136, 0.624], q = 2.027). The increase in PO PRN medications used from 2023 to 2024 was also not statistically significant (mean difference = 0.392 orders/day, 95% CI [-0.005, 0.790], q = 3.277), but by a small margin. This indicates the 2024 group in Unit A was associated with increased oral sedation and antipsychotic control, particularly when compared to 2022, while no trend occurred in Unit B. Please see **Figure 1** below comparing the average number of PO PRN orders per day for each cohort. Please refer to **Tables 2** and **3** at the end of the manuscript for the results of ANOVA and *post-hoc* analysis of PO PRN orders.

**Table 2:** PO PRN medications ordered per day in Unit A with ANOVA and *post-hoc* analysis.

Summary						
Groups	Sum	Average	Variance			
2022	59	0.756	1.070			
2023	78	1.000	1.143			
2024	110	1.392	1.165			
ANOVA						
Source of Variation	SS	df	MS	F	P-value	F crit
Between Groups	16.180	2	8.090	7.185	0.001	3.035
Within Groups	261.207	232	1.126			
Total	277.387	234				
Tukey-Kramer HSD (critical q value = 3.337)						
	Difference between groups	n1	n2	SE	q	
2024 vs 2023	0.392	79	78	0.120	3.277	
2024 vs 2022	0.636	79	78	0.120	5.310	
2023 vs 2022	0.244	78	78	0.120	2.027	

**Table 3:** PO PRN medications ordered per day in Unit B with ANOVA and *post-hoc* analysis.

Summary						
Groups	Sum	Average	Variance			
2022	734	8.738	>25.786			
2023	734	8.738	>27.425			
2024	736	8.659	>16.061			
ANOVA						
Source of Variation	SS	df	MS	F	P-value	F crit
Between Groups	0.355	2	0.177	0.008	0.992	3.032
Within Groups	5765.582	250	23.062			
Total	5765.937	252				
Tukey-Kramer HSD (critical q value = 3.335)						
	Difference between groups	n1	n2	SE	q	
2024 vs 2023	0.079	85	84	0.522	0.152	
2024 vs 2022	0.079	85	84	0.522	0.152	
2023 vs 2022	0.000	84	84	0.524	0.000	

### 3.3 IM PRN orders

**Figure 2:**
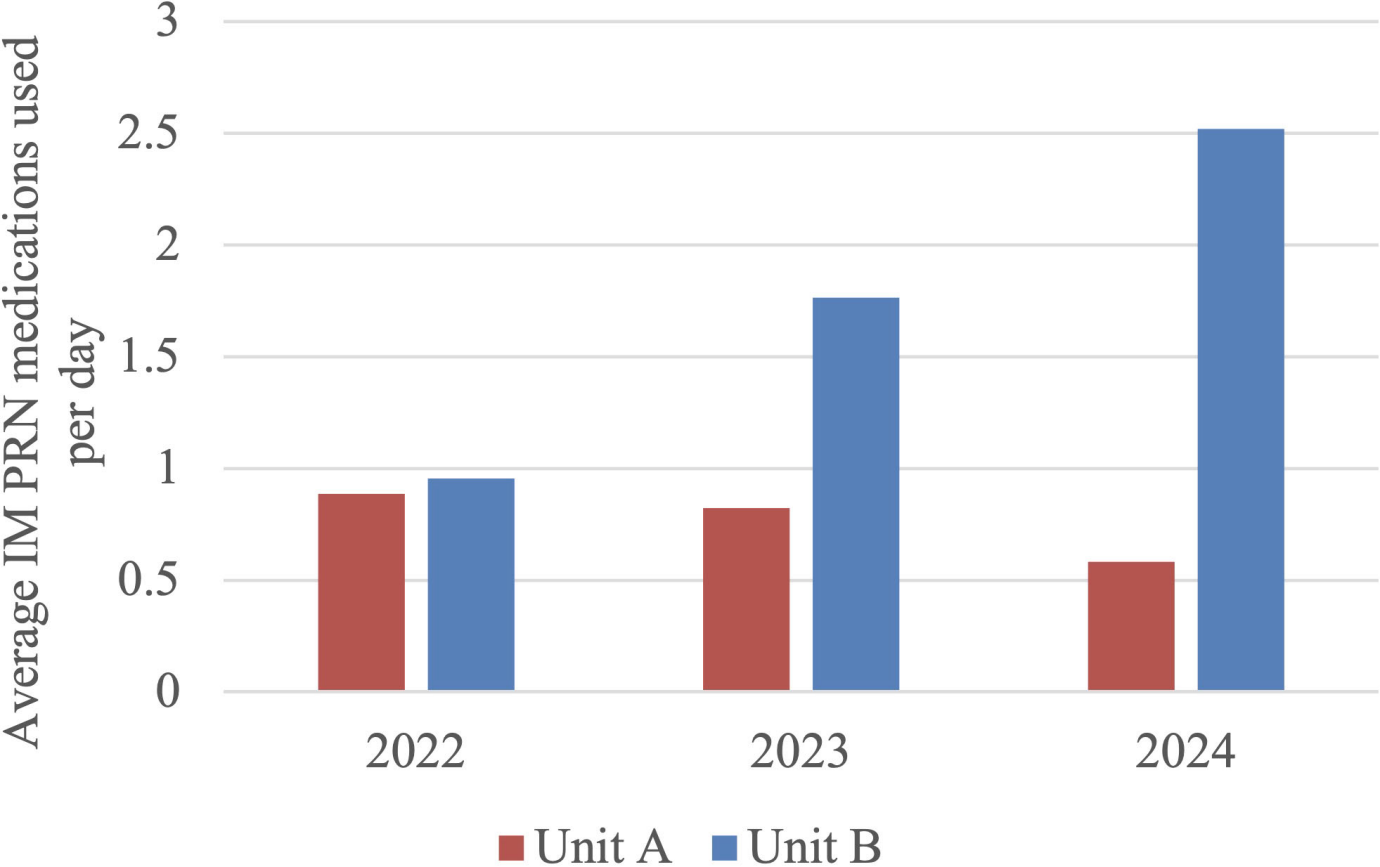
Average IM PRN medication ordered per day in Unit A and Unit B. In Unit A, no trend was observed. In Unit B, the 2023 and 2024 groups both saw increased IM PRN orders compared to 2022.

For IM medication orders, ANOVA analysis found no significant difference in Unit A (F = 1.530, p = 0.219). However, there was statistically significant differences between groups in Unit B (F = 10.554, p < 0.001). The Tukey-Kramer HSD test (critical q value = 3.335) demonstrated that there were statistically significant increases in IM medication orders when comparing 2022 vs 2023 (mean difference = 0.810 orders/day, 95% CI [0.354, 1.266], q = 3.350) and 2022 vs 2024 (mean difference = 1.565 orders/day, 95% CI [1.109, 2.021], q = 6.497), but no significant difference when comparing 2023 vs 2024 (mean difference = 0.756 orders/day, 95% CI [0.298, 1.214], q = 3.137). On Unit B, average daily IM medication orders showed a statistically significant increase from 2022 to 2023 (from 0.952 to 1.762 orders/day) before the outdoor space closure, and then a further increase to 2.518 orders/day in 2024. This suggests a pre-existing upward trend in IM PRN use on Unit B, which may be indicative of other unmeasured factors. Please see **Figure 2** below for the average number of IM PRN orders per day for each cohort. Please refer to **Tables 4** and **5** at the end of the manuscript for the results of ANOVA and *post-hoc* analysis of IM PRN orders.

**Table 4:** IM PRN medications ordered per day in Unit A with ANOVA and post-hoc analysis.

Summary						
Groups	Sum	Average	Variance			
2022	69	0.885	1.844			
2023	64	0.821	1.110			
2024	46	0.582	0.964			
ANOVA						
Source of Variation	SS	df	MS	F	P-value	F crit
Between Groups	3.991	2	1.996	1.530	0.219	3.035
Within Groups	302.664	232	1.305			
Total	306.655	234				
Tukey-Kramer HSD (critical q value = 3.337)						
	Difference between groups	n1	n2	SE	q	
2024 vs 2023	0.238	79	78	0.129	1.848	
2024 vs 2022	0.302	79	78	0.129	2.345	
2023 vs 2022	0.064	78	78	0.129	0.496	

**Table 5:** IM PRN medications ordered per day in Unit B with ANOVA and post-hoc analysis.

Summary						
Groups	Sum	Average	Variance			
2022	80	0.952	2.552			
2023	148	1.762	3.919			
2024	214	2.518	8.205			
ANOVA						
Source of Variation	SS	df	MS	F	P-value	F crit
Between Groups	103.539	2	51.770	10.554	< 0.001	3.032
Within Groups	1226.271	250	4.905			
Total	1329.810	252				
Tukey-Kramer HSD (critical q value = 3.335)						
	Difference between groups	n1	n2	SE	q	
2024 vs 2023	0.756	85	84	0.241	3.137	
2024 vs 2022	1.565	85	84	0.241	6.497	
2023 vs 2022	0.810	84	84	0.242	3.350	

### 3.4 Seclusion and restraint orders

**Figure 3:**
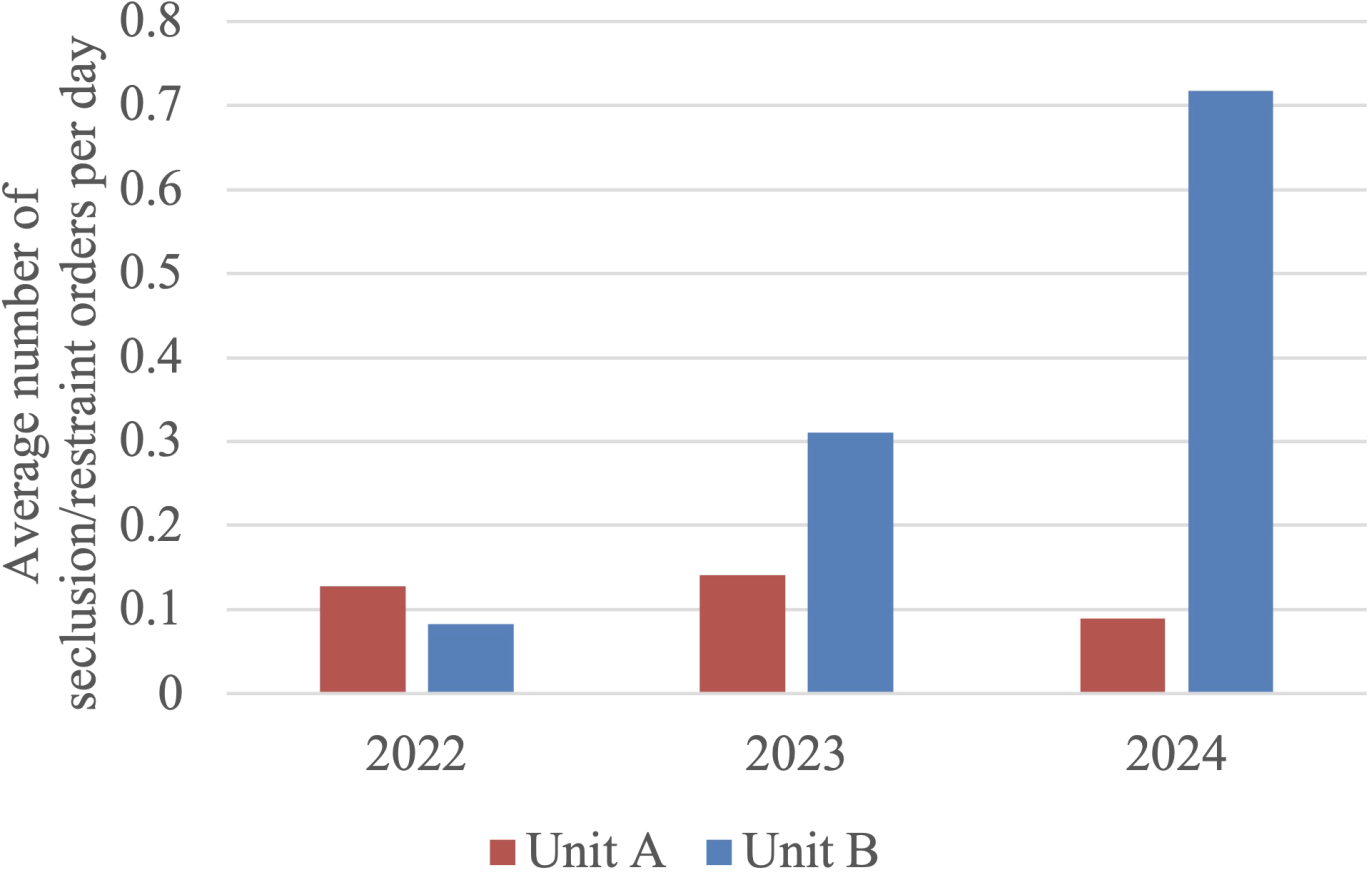
Average number of seclusion and/or restraint orders per day in Unit A and Unit B. In Unit A, no trend was observed. In Unit B, seclusion and restraint orders were greater in 2024 compared to prior years.

When examining use of seclusion and restraints, ANOVA analysis found no statistical difference between groups in Unit A (F = 0.373, p = 0.689) but did find significant differences in Unit B (F = 7.198, p < 0.001). The Tukey-Kramer HSD test here specifically found significance when comparing 2022 vs 2024 (mean difference = 0.634 orders/day, 95% CI [0.351, 0.918], q = 5.291) and 2023 vs 2024 (mean difference = 0.408 orders/day, 95% CI [0.125, 0.691], q = 3.404), but not when comparing 2022 vs 2023 (mean difference = 0.226 orders/day, 95% CI [-0.057, 0.510], q = 1.881). While the average daily orders for seclusion and restraint on Unit B increased substantially from 0.083 in 2022 to 0.310 in 2023, and further to 0.718 in 2024, the difference between 2022 and 2023 was not statistically significant. The statistically significant increases were observed primarily when comparing 2024 to previous years, indicating that 2024 was associated with increased use of seclusion and restraint, but only in Unit B. Please see **Figure 3** below for the average number of seclusion and/or restraint orders per day. Please refer to **Tables 6** and **7** at the end of the manuscript for the results of ANOVA and *post-hoc* analysis of restraint and seclusion orders.

**Table 6:** Seclusion and/or restraint ordered per day in Unit A with ANOVA and post-hoc analysis.

Summary						
Groups	Sum	Average	Variance			
2022	10	0.128	0.113			
2023	11	0.141	0.175			
2024	7	0.089	0.184			
ANOVA						
Source of Variation	SS	df	MS	F	P-value	F crit
Between Groups	0.117	2	0.059	0.373	0.689	3.035
Within Groups	36.546	232	0.158			
Total	36.664	234				
Tukey-Kramer HSD (critical q value = 3.337)						
	Difference between groups	n1	n2	SE	q	
2024 vs 2023	0.052	79	78	1.719	0.030	
2024 vs 2022	0.040	79	78	1.719	0.023	
2023 vs 2022	0.013	78	78	1.725	0.007	

**Table 7:** Seclusion and/or restraint ordered per day in Unit B with ANOVA and post-hoc analysis.

Summary						
Groups	Sum	Average	Variance			
2022	7	0.083	0.174			
2023	26	0.310	1.011			
2024	61	0.718	2.443			
ANOVA						
Source of Variation	SS	df	MS	F	P-value	F crit
Between Groups	17.483	2	8.741	7.198	0.001	3.032
Within Groups	303.593	250	1.214			
Total	321.075	252				
Tukey-Kramer HSD (critical q value = 3.335)						
	Difference between groups	n1	n2	SE	q	
2024 vs 2023	0.408	85	84	0.120	3.404	
2024 vs 2022	0.634	85	84	0.120	5.291	
2023 vs 2022	0.226	84	84	0.120	1.881	

### 3.5 Constant observation orders

**Figure 4:**
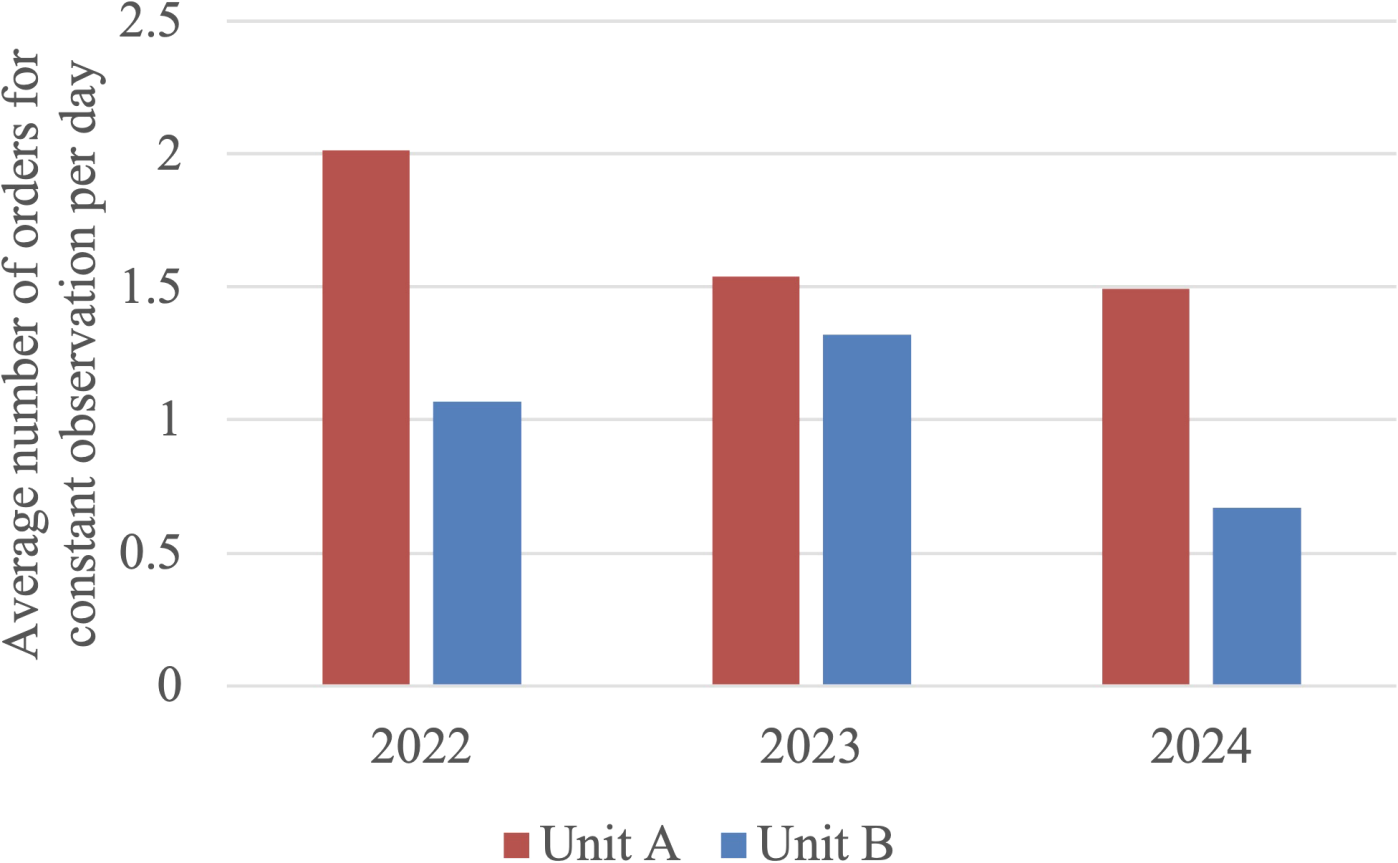
Average number of constant observation orders per day in Unit A and Unit B. In Unit A, constant observation was utilized significantly more in 2022 compared to 2023 or 2024. In Unit B, constant observation use in 2024 was decreased compared to prior years.

ANOVA analysis found statistical significance in both Unit A (F = 5.248, p = 0.006) and Unit B (F = 12.900, p < 0.001). In Unit A, the Tukey-Kramer HSD test (critical q value = 3.337) found significant differences when comparing 2022 vs 2023 (mean difference = 0.474 orders/day, 95% CI [0.106, 0.843], q = 3.772) and 2022 vs 2024 (mean difference = 0.519 orders/day, 95% CI [0.151, 0.887], q = 4.142), but not 2023 vs 2024 (mean difference = 0.045 orders/day, 95% CI [-0.324, 0.414], q = 0.357). These results indicate that average constant observation rates were significantly higher in 2022 compared to the later years. In Unit B, the Tukey Kramer HSD test (critical q value = 3.335) did find significant differences when comparing 2022 vs 2024 (mean difference = 0.401 orders/day, 95% CI [0.207, 0.595], q = 4.383) and 2023 vs 2024 (mean difference = 0.651 orders/day, 95% CI [0.457, 0.845], q = 7.117), but not when comparing 2022 vs 2023 (mean difference = 0.250 orders/day, 95% CI [0.055, 0.445], q = 2.726). The trends notably demonstrate decreased constant observation orders in 2024 compared to the previous years. Please see **Figure 4** below for the average number of patients under constant observation per day. Please refer to **Tables 8** and **9** at the end of the manuscript for the results of ANOVA and *post-hoc* analysis of constant observation orders.

**Table 8:** Constant observation orders per day in Unit A with ANOVA and post-hoc analysis.

Summary						
Groups	Sum	Average	Variance			
2022	157	2.013	1.831			
2023	120	1.538	0.849			
2024	118	1.494	1.022			
ANOVA						
Source of Variation	SS	df	MS	F	P-value	F crit
Between Groups	12.945	2	6.473	5.248	0.006	3.035
Within Groups	286.119	232	1.233			
Total	299.064	234				
Tukey-Kramer HSD (critical q value = 3.337)						
	Difference between groups	n1	n2	SE	q	
2024 vs 2023	0.045	79	78	0.125	0.357	
2024 vs 2022	0.519	79	78	0.125	4.142	
2023 vs 2022	0.474	78	78	0.126	3.772	

**Table 9:** Constant observation orders per day in Unit B with ANOVA and post-hoc analysis.

Summary						
Groups	Sum	Average	Variance			
2022	90	1.071	0.935			
2023	111	1.321	0.462			
2024	57	0.671	0.724			
ANOVA						
Source of Variation	SS	df	MS	F	P-value	F crit
Between Groups	18.232	2	9.116	12.900	0.000	3.032
Within Groups	176.669	250	0.707			
Total	194.901	252				
Tukey-Kramer HSD (critical q value = 3.335)						
	Difference between groups	n1	n2	SE	q	
2024 vs 2023	0.651	85	84	0.091	7.117	
2024 vs 2022	0.401	85	84	0.091	4.383	
2023 vs 2022	0.250	84	84	0.092	2.726	

## 4 Discussion

This retrospective cohort study investigated the differential correlation of outdoor space accessibility with PRN medication use and restrictive interventions across two distinct acute psychiatric units: a Geriatric Psychiatry Unit (Unit A) and a General Psychiatry Unit (Unit B). The analysis specifically compared periods of outdoor space inaccessibility (2024) with periods of accessibility (2022 and 2023) across both units.

On the General Psychiatry Unit (Unit B), a significant association was found between the inaccessibility of outdoor space and higher rates of interventions. During the period when the outdoor space was inaccessible (2024), there were significantly higher rates of intramuscular (IM) as-needed (PRN) medication usage as well as greater seclusion and restraint orders compared to when the outdoor space was accessible. However, it is crucial to note the pre-existing trends in Unit B: IM PRN use showed a statistically significant increase between 2022 and 2023, prior to the outdoor space closure, suggesting other factors may have already been driving this increase. While seclusion and restraint also showed a visually substantial increase from 2022 to 2023, this difference was not statistically significant. The significant increases in both IM PRN and seclusion/restraint were primarily observed when comparing 2024 to previous years, suggesting that the loss of outdoor access may have exacerbated these trends or introduced additional pressure. Unit B also saw a decrease in constant observation orders compared to when the outdoor space was accessible (2022-2023). This decrease in constant observation was likely related to a hospital-wide leadership strategy to decrease the total number of constant observations within the hospital. Notably, no significant difference was found in oral (PO) PRN medication use in Unit B.

In contrast, findings from the Geriatric Psychiatry Unit (Unit A) presented a different pattern. Here, outdoor space inaccessibility was associated with a significant increase exclusively in PO PRN medication use. However, IM PRN use, seclusion and restraint, and CO did not show any statistically significant increase directly attributable to the lack of outdoor access. It is important to note that a broader hospital-wide trend indicated significantly lower CO rates in 2023 and 2024 compared to 2022 on Unit A, suggesting that this overall decline in CO is likely a result of systemic quality improvement initiatives rather than a direct consequence of outdoor space accessibility or an indicator of reduced patient agitation.

These findings suggest a differential correlation of outdoor space accessibility with intervention use across distinct patient populations. The lack of accessible outdoor space appears to influence the general adult psychiatric population (Unit B) more broadly, potentially contributing to increased reliance on more restrictive interventions including IM PRN, seclusion, and restraint. This heightened need for intervention may be attributed to reduced opportunities for patients to engage in self-regulation through fresh air exposure, personal space, and physical distancing from unit stressors. Additionally, staff perceptions of limited environmental de-escalation options might contribute to a higher propensity for intervention use, as qualitative studies on environmental factors in aggression management have suggested (12). Conversely, the geriatric population (Unit A) primarily experienced an increase in PO PRN use when outdoor space was inaccessible, while more restrictive measures remained largely unaffected. This differential response could be due to the inherent lower mobility, reduced activity levels, and general frailty typical of geriatric patients, which naturally limits the potential for behaviors necessitating such interventions.

The hypothesis that outdoor space access reduces the need for interventions received differential support from across the two units. For Unit B, the increases in IM PRN, seclusion, and restraint during the period of outdoor space inaccessibility align with our hypothesis, suggesting that environmental factors may play a role in behavioral regulation for a general adult psychiatric population. However, the existing upward trends in IM PRN use on Unit B from 2022 to 2023, prior to the outdoor space closure, strongly suggest that other unmeasured factors were already contributing to an increase in intervention rates. This complicates the interpretation of a direct causal link solely to outdoor space inaccessibility for IM PRN. Yet, the further increase in 2024 may indicate an additional influence. Similarly, the visually substantial, albeit not statistically significant, increase in seclusion and restraint on Unit B from 2022 to 2023 further highlights the likelihood of multiple contributing factors.

The absence of a similar increase in more restrictive measures in Unit A, with only an increase in PO PRN, underscores the importance of patient characteristics (e.g., mobility, cognitive status) in modulating the influence of environmental stressors. This resonates with prior literature suggesting that environmental interventions should be tailored to specific patient populations (4). The observed hospital-wide initiative for CO reduction serving as a confounder for that specific outcome, particularly in Unit A, highlights the challenge of isolating single environmental factors in real-world clinical settings, where multiple quality improvement efforts may be simultaneously active. This aligns with the multifactorial nature of agitation and aggression in psychiatric settings as discussed by Weltens et al., where clinician, healthcare system, and environmental factors all interplay (1). Given these complexities and the pre-existing trends, the results regarding the effect of outdoor space on coercive measures remain somewhat inconclusive and require cautious interpretation.

### 4.1 Limitations

This study has several important limitations that warrant critical discussion. First, its retrospective nature limits the control over variables and introduces the potential for information bias. Data relied solely on electronic medical records, and while these are routinely collected, their primary purpose is clinical care, not research. Inconsistencies or nuances in documentation practices over time or between units could influence results.

Second, the outcomes were measured solely by proxy measures (PRN medications, seclusion, restraint, and constant observation). While these interventions are commonly used in the psychiatric literature as indicators of acute behavioral disturbance and patient management, they do not directly measure the actual frequency, severity, or type of aggressive behaviors. Staff decisions to employ these interventions are multifactorial, influenced by patient presentation, staff training, unit culture, and available resources, which could introduce measurement bias. Direct observational measures of aggression, which have been employed in other contexts, would provide more granular and specific insights (13).

Third, a major assumption of this quasi-experimental design was that no other significant confounding changes occurred in unit operations, staffing levels, treatment regimens, or patient populations during the study period, other than the outdoor space accessibility. This assumption is rarely, if ever, perfectly true in real-world clinical settings. While demographic and diagnostic characteristics appeared generally stable across the yearly cohorts within each unit (**Table 1**), we could not control for unmeasured confounders such as daily staffing levels, staff-patient ratios, staff experience, specific therapeutic programs offered, or the severity of illness of admitted patients beyond general diagnostic categories. For instance, an unmeasured change in staff training on de-escalation techniques could influence intervention rates. The finding of a hospital-wide initiative to reduce constant observation orders explicitly indicates a breakdown of this assumption for at least one outcome, highlighting the potential for other unmeasured institutional quality improvement initiatives or policy changes to confound results. Furthermore, the observed upward trend in IM PRN use on Unit B from 2022 to 2023, prior to the loss of outdoor access, suggests the presence of unidentified factors influencing intervention rates, making it difficult to isolate the precise impact of outdoor space inaccessibility alone. It is also possible that the observed effects could be partially due to a simple reduction in available space, leading to increased overcrowding on the ward, rather than specifically the lack of outdoor elements.

Fourth, the generalizability of these findings may be limited. The study was conducted at a single free-standing psychiatric hospital in the United States, and the specific patient populations (geriatric vs. general adult acute care) and unit designs may not be representative of other psychiatric facilities globally. The potential influence of outdoor space might also vary depending on the specific design of the outdoor area (e.g., size, presence of nature, furniture, perceived safety), its regulations, seasonal weather changes, and the cultural context.

## 5 Conclusions

The findings suggest that the temporary loss of outdoor space was associated with increased PRN medication use and restrictive interventions in the acute care inpatient psychiatric unit, particularly affecting the more active and mobile general adult population. The geriatric population showed a milder response, primarily in PO PRN use. This result indicates that the environment is an important factor to consider alongside other therapeutic interventions when managing patient agitation and behavior in the psychiatric unit. The limitations of this study, including the reliance on proxy measures and the potential for unmeasured confounders, call for future investigation, where the measurement of aggression is done directly, and where more robust statistical controls for confounding variables are employed.

In conclusion, accessible outdoor space appears to be a critical environmental factor associated with the use of restrictive interventions in acute psychiatric settings, with its influence being particularly salient within general adult populations. These results underscore the importance of integrating accessible outdoor spaces into psychiatric unit design and operational planning to promote patient well-being and potentially minimize the need for coercive measures.

## Data Availability

The original contributions presented in the study are included in the article/supplementary material. Further inquiries can be directed to the corresponding author.
