# Correction: Healing spaces: a retrospective cohort study on the effect of outdoor spaces in psychiatric inpatient units on PRN medication use, seclusion/restraints, and constant observation

**DOI:** 10.3389/fpsyt.2026.1796681

**Published:** 2026-02-06

**Authors:** Kevin Liu, Ema Saito, Howard Linder

**Affiliations:** 1 Donald and Barbara Zucker School of Medicine at Hofstra/Northwell, Hempstead, NY, United States; 2 Department of Psychiatry, The Zucker Hillside Hospital, Northwell Health, Glen Oaks, NY, United States

**Keywords:** acute psychiatry, constant observation, outdoor space, PRN medication, restraint, seclusion

In the published article, there was an error. In the **Abstract**, *Results*, constant observation (CO) in unit B was incorrectly described as having increased with outdoor space inaccessibility in 2024, rather than decreased. CO in unit A was incorrectly described as having no change with outdoor space inaccessibility, when there was actually a decrease when compared to 2022, although not to 2023. This paragraph previously stated:

“On unit B, outdoor space inaccessibility was significantly associated with higher IM PRN use (mean difference = 1.57 orders/day, 95% CI [0.81, 2.33]), seclusion and restraint (mean difference = 0.63 orders/day, 95% CI [0.35, 0.91]), and CO (mean difference = 0.40 orders/day, 95% CI [0.17, 0.63]), with no significant change in PO PRN. On unit A, outdoor space inaccessibility was associated with an increase in PO PRN use (mean difference = 0.64 orders/day, 95% CI [0.26, 1.02]), but IM PRN, seclusion and restraint, and CO did not show a statistically significant change.”

The corrected paragraph appears below:

“On unit B, outdoor space inaccessibility was significantly associated with increased IM PRN use (mean difference = 1.57 orders/day, 95% CI [0.81, 2.33]), increased seclusion and restraint (mean difference = 0.63 orders/day, 95% CI [0.35, 0.91]), and decreased CO (mean difference = 0.40 orders/day, 95% CI [0.17, 0.63]), with no significant change in PO PRN. On unit A, outdoor space inaccessibility was associated with an increase in PO PRN use (mean difference = 0.64 orders/day, 95% CI [0.26, 1.02]), while IM PRN, seclusion and restraint, and CO did not show any statistically significant increase.”

The original article has been updated.

